# A clinical follow-up study of premature thelarche in infants under the age of two

**DOI:** 10.1186/1687-9856-2013-S1-P129

**Published:** 2013-10-03

**Authors:** Yingmin Wang, Li Liang, Yanlan Fang, Junfen Fu, Guanping Dong, Chunlin Wang

**Affiliations:** 1 Department of Endocrinology, Children's Hospital Affiliated to Zhejiang University Medicale College, Hangzhou 310003, China

**Keywords:** Premature thelarche, Prevalence, Follow-up, Infancy

## Objective

Assess the clinical status and natural course of premature thelarche in infants under the age of 2 years as well as analyze the predictive factors for thelarche regression.

## Method

Through Hospital Information System, analyze the hospital-based prevalence of premature thelarche in infants under the age of 2 years in recent 3 years as well as analyze the follow-up databases of clinical features and laboratory data in 890 patients under 2 years old from October 2009 to September 2010.

## Result

1.Hospital-based prevalence in infants under the age of 2 years in 2009, 2010 and 2011 is 0.26‰, 0.40‰, 0.39‰ relatively. On average it's 0.35‰. Period from July to September in every year is the peak time of doctor visiting. 2. Most (99.8%)of infants with premature thelarche are manifested as isolated premature thelarche, while only 0.2% are represented as peripheral precocious puberty. 3. Among the patients under the age of 2 years with isolated premature thelarche 89.5% of them get a regression between the age of 2 and 3 years( The average of regression age is 17±5.8 months), 10.5% of them do not make recovery by the age of 3 years and even some (0.4%) turn into central precocious puberty(CPP). 4. The two influencing factors of breast regression are breast size by the first time of doctor visiting and whether basal estrogen is high or not.

## Conclusion

Premature thelarche in infants under the age of 2 is not a rare disease. Remaining of premature thelarche is probably associated with minipuberty and environmental estrogen disruptors. Premature thelarche in the majority manifested as a self-limited condition. Meanwhile, follow-ups at regular intervals to those remaining symptoms above 2 years old are needed.

